# Supplementary material for: Novel anti-PTEN C2 domain monoclonal antibodies to analyse the expression and function of PTEN isoform variants
Source: PLoS One. 2023 Aug 1;18(8):e0289369. doi: 10.1371/journal.pone.0289369 (PMC10393154; doi:10.1371/journal.pone.0289369)

Figure 1A

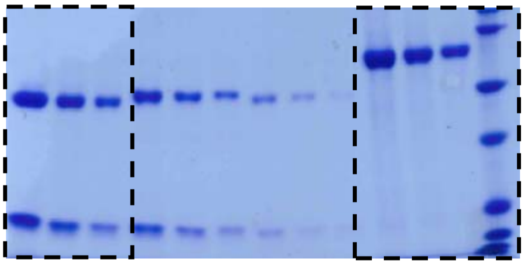

Figure 1C

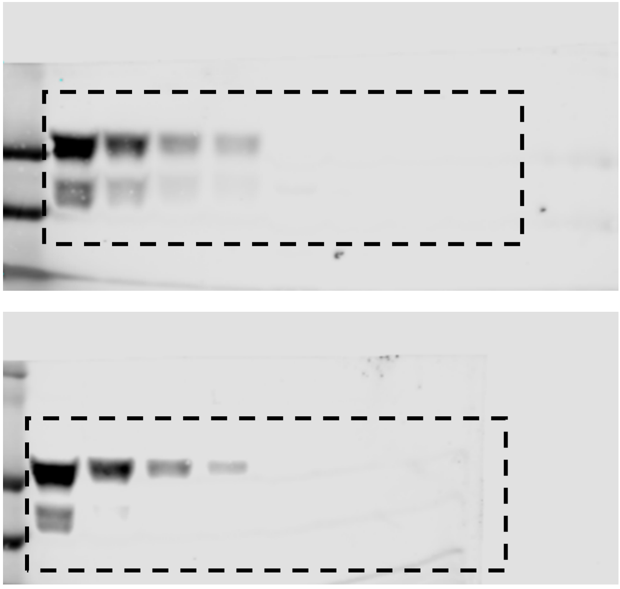

Uncropped gels and blots for Figure 1

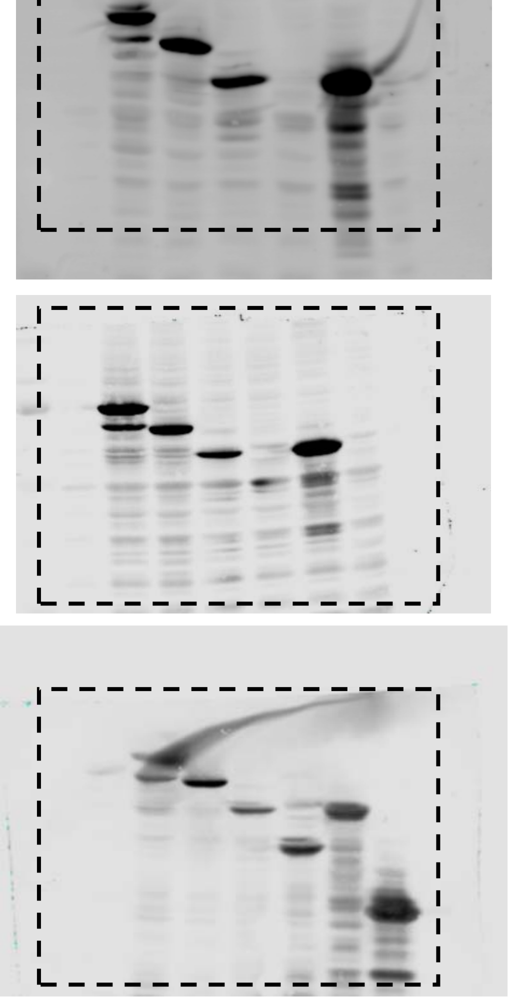

Figure 2D

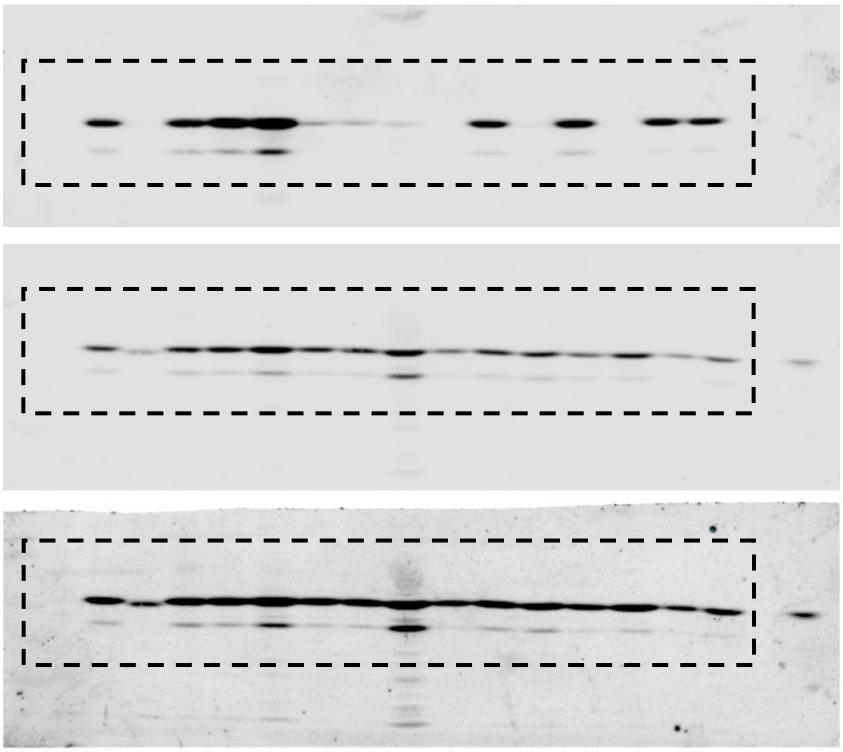

Figure 2B

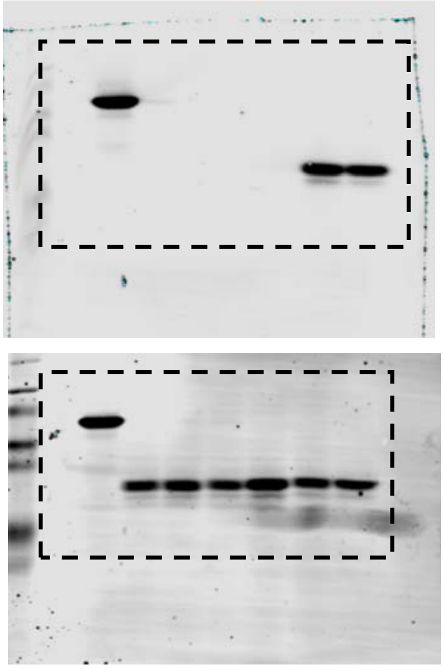

Figure 2E

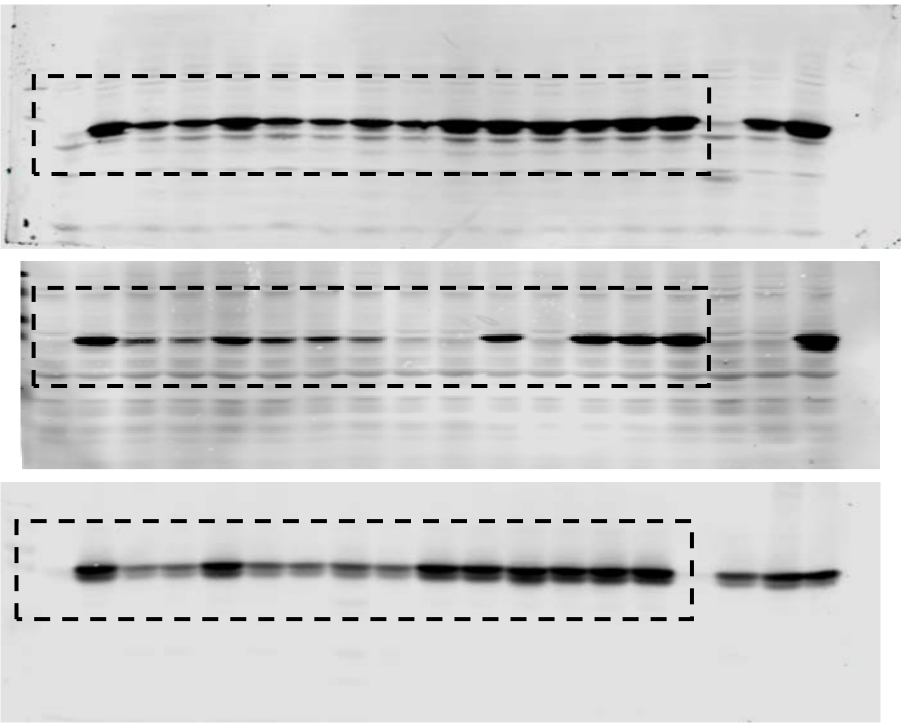

Figure 2C

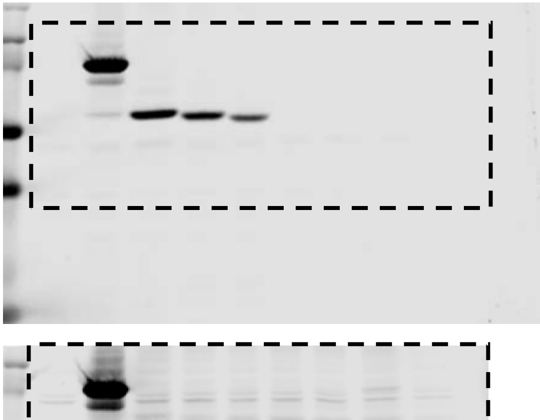

Figure 5A

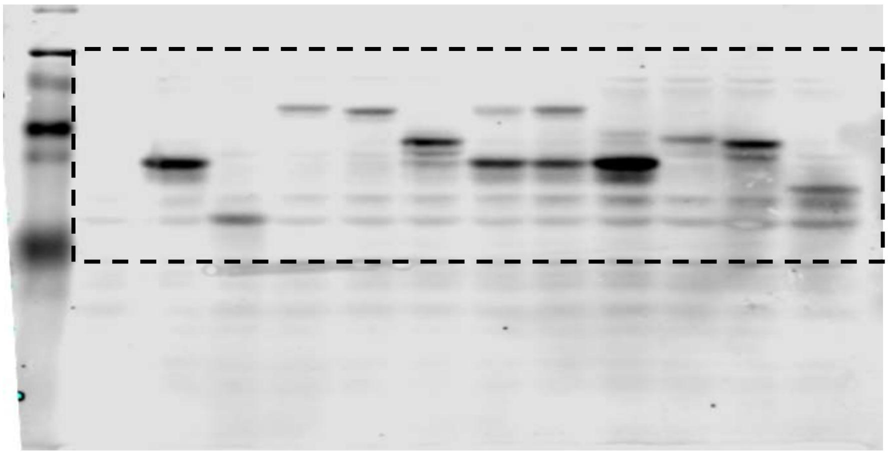

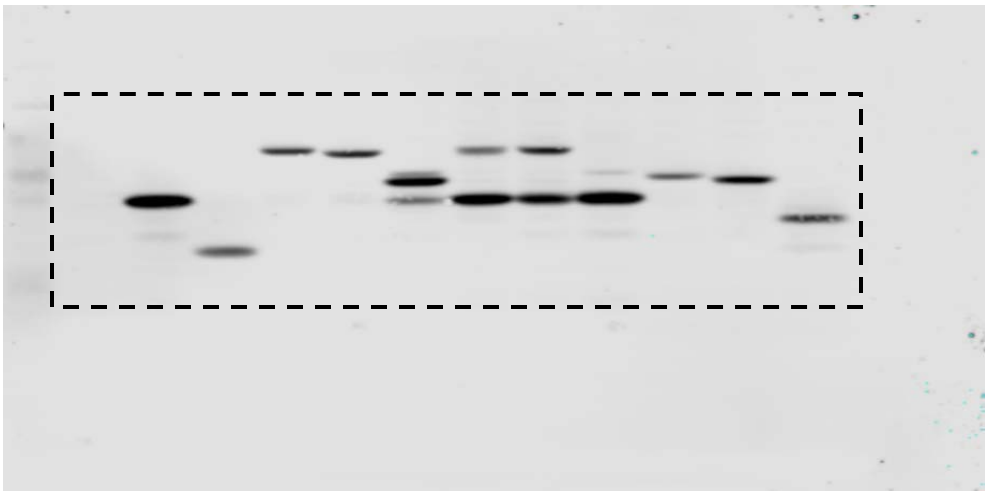

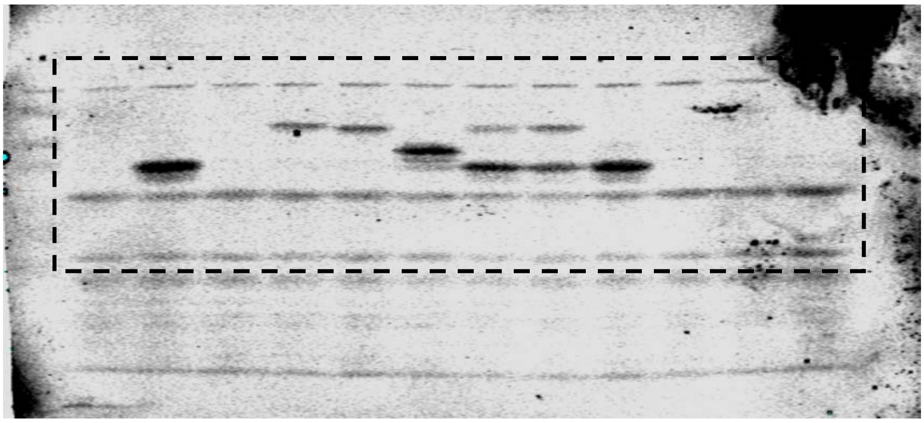

Uncropped blots for Figure 5A

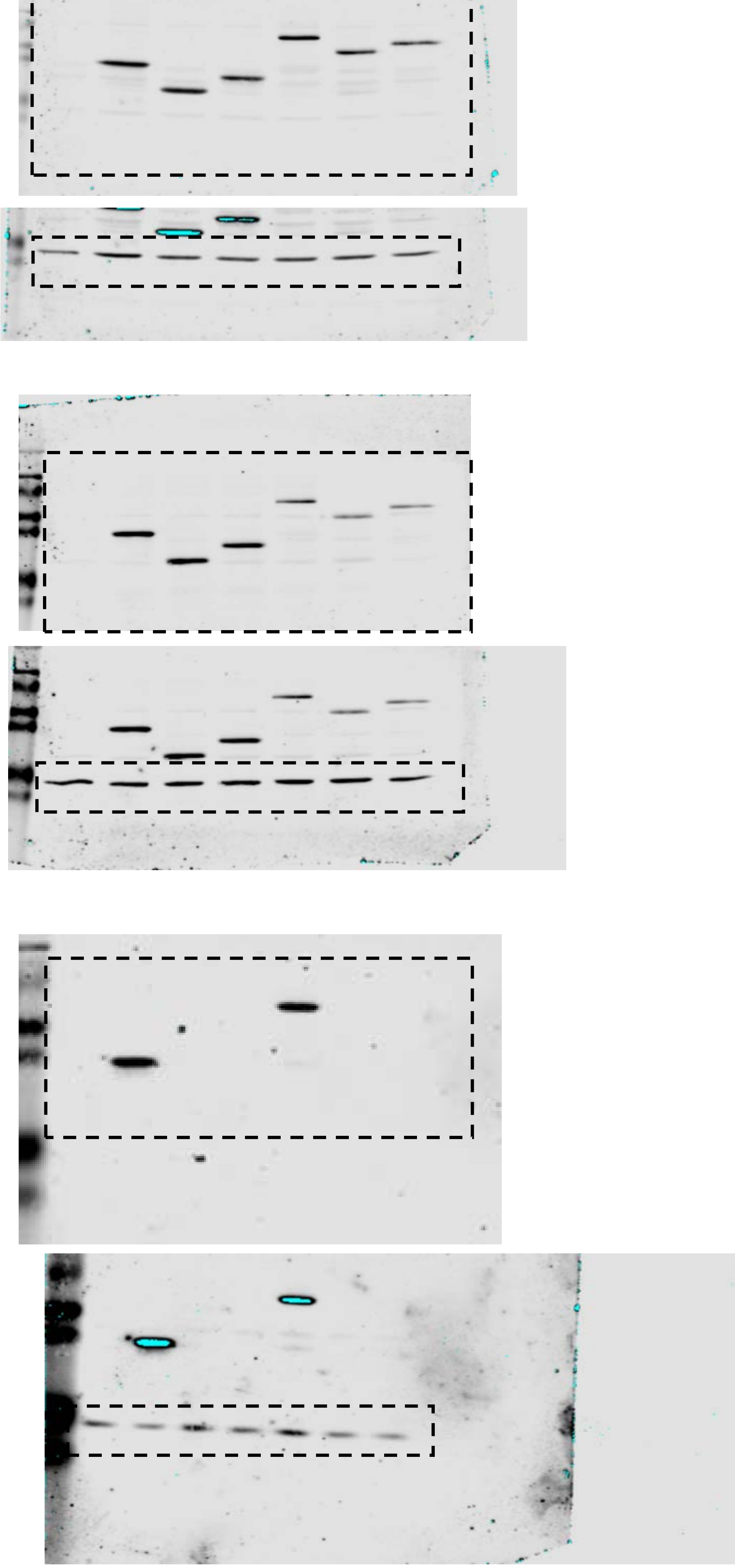

Uncropped blots for Figure 5B

Figure 6A

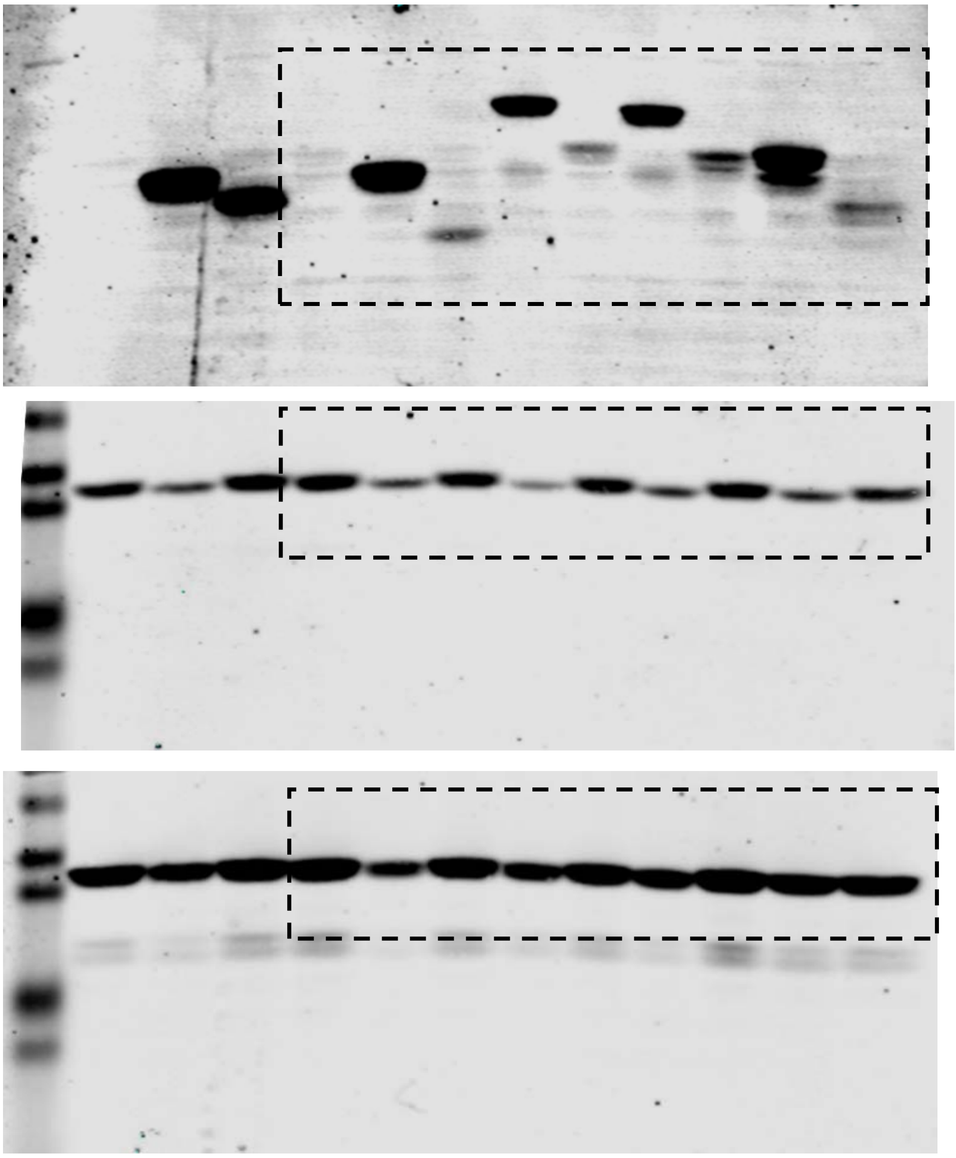

Uncropped blots for Figure 6A

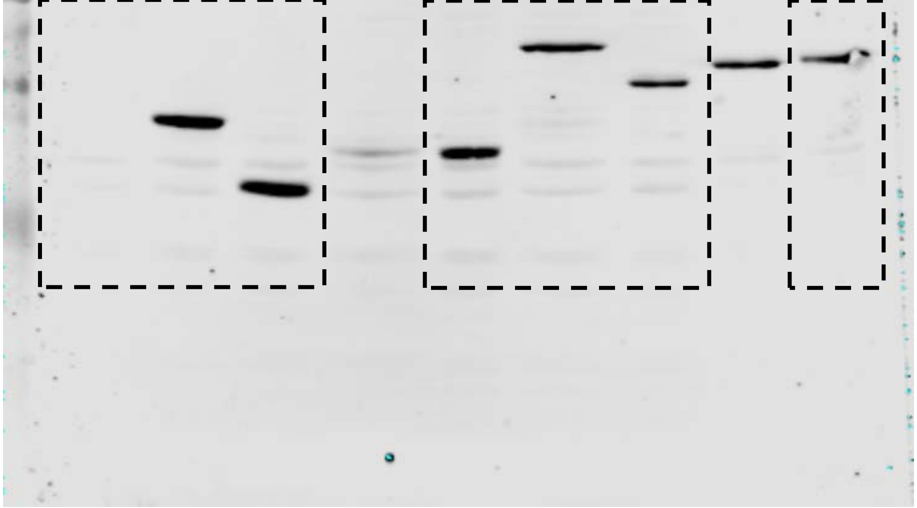
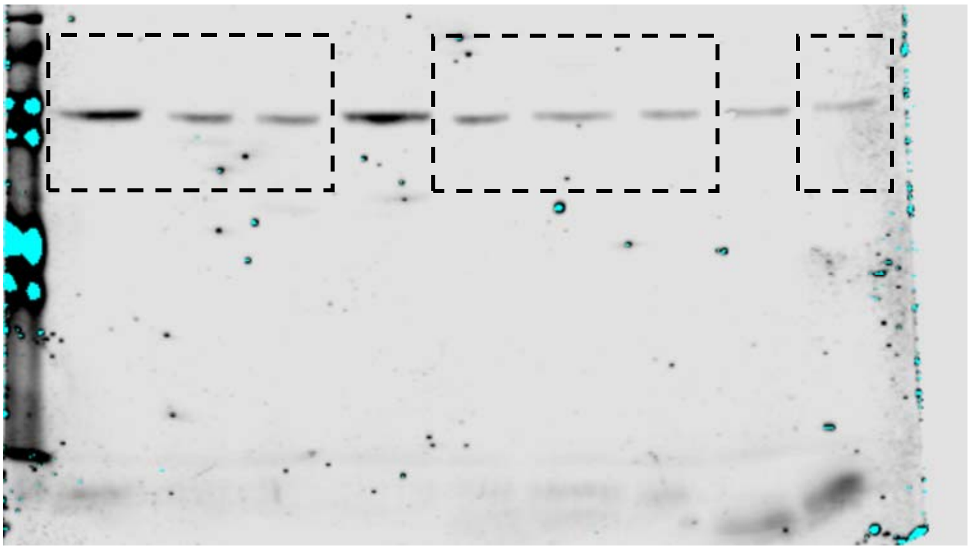
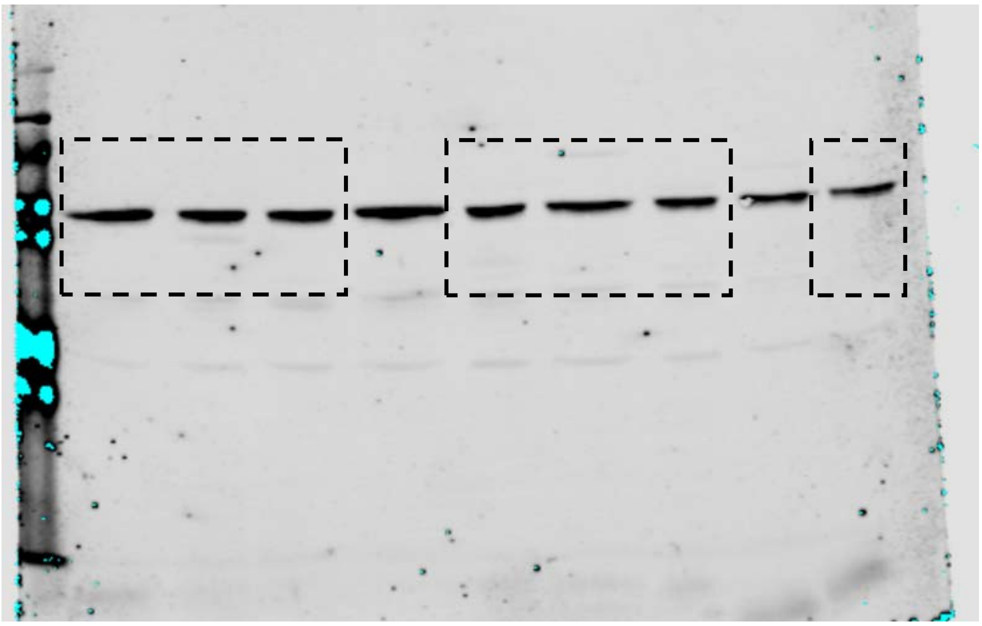
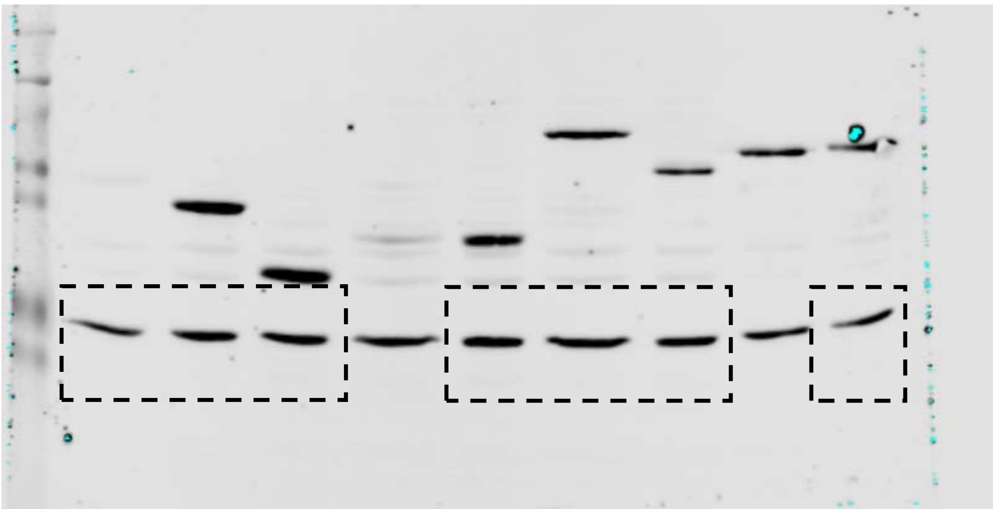

Supplement: S1 Raw images — (PDF) [file pone.0289369.s001.pdf]
