# Supplementary material for: Novel anti-PTEN C2 domain monoclonal antibodies to analyse the expression and function of PTEN isoform variants
Source: PLoS One. 2023 Aug 1;18(8):e0289369. doi: 10.1371/journal.pone.0289369 (PMC10393154; doi:10.1371/journal.pone.0289369)
Supplement: Attachment — Submitted filename: Torices.Response to reviewers.15jun23.doc [file pone.0289369.s002.doc]

Dr. Avaniyapuram Kannan Murugan
Academic Editor, PLOS ONE

15 June 2023

Dear Dr. Murugan,

Thank you very much for your letter from 5 May 2023 regarding our manuscript PONE-D-23-09497. We appreciate and thank to the referees for their time and constructive comments, and to you for giving us the opportunity to submit a revised version of the manuscript. We have revised the manuscript addressing all the comments and specific points of the reviewers, and we submit here a new manuscript version for your consideration.

As a summary of the major changes, we present now a new Figure 1 which includes comparative IHC staining of the new anti-PTEN BA226 and the commercial anti-PTEN 6H2.1 mAb (Figure 1E). Also, a new Figure 5 is presented that includes the anti-GAPDH control blots requested (Figure 5B), as well as novel immunofluorescence experiments to show detection of nuclear PTEN by the BA226 mAb (novel Figure 5C).

Following are our answers, point by point, to the reviewers’ comments (in red and italics, reviewers’ comments; in black, our answers).

**Reviewer #1**

Specific points from Reviewer #1:

*I would recommend expand the analysis of human samples (as those shown in Fig 1E) by immunohistochemistry including tumors (prostate is a good example) of known status of PTEN gene (different mutations, deletions, etc.) using the BA226 mAb. This could maximize the use of the antibody in the clinical practice.*

We agree with the comment of the reviewer, but unfortunately we do not have available at this moment clinical samples with known status of PTEN gene to perform IHC analysis. Instead, we show now in the novel Figure 1E the comparative staining of the novel anti-PTEN BA226 mAb and the commercial anti-PTEN mAb more frequently used in the clinical practice (6H2.1 mAb) in negative and positive prostate adenocarcinoma specimens.

*It would be worth performing immunofluorescence analysis of endogenous PTEN using different cell lines, again with different known PTEN status. Is this antibody reacting similarly with the different localizations of endogenous PTEN (mainly nuclear and cytoplasm)? Given that the functional roles of PTEN may differ between these two localizations this aspect could also be relevant*

To the best of our knowledge, there are no cell lines displaying specifically distinct PTEN nucleus/cytoplasm subcellular localization. Since we consider the observation of the reviewer pertinent, we now show in the novel Figure 5C the immunofluorescence staining by BA226 mAb of a PTEN nuclear form (PTEN Y379X) ectopically expressed in COS-7 cells.

*One potential interesting aspect is to know whether this antibody reacts also with other species.*

The amino acid sequence of PTEN is highly conserved between species. In mammals, conservation is almost 100%, with some mammalian species used as model organisms displaying one amino acid substitution (change of Thr398 in human to Ser398 in mouse or rat; or change of Ser294 in human to Gly294 in pig). Interestingly, other more distant vertebrate model organisms, such as chicken (Gallus gallus), clawed frog (Xenopus tropicalis), or zebrafish (Danio rerio) display local amino acid sequence differences in PTEN with respect to human which affect to the epitopes recognized by the anti-PTEN BA226 and 425A described in the manuscript. A sentence has been included in the Discussion section on how this could affect the recognition of PTEN by the BA226 and 425A antibodies in those species, and on the suitability of these antibodies to monitor heterologous expression of human PTEN in cells from these non-mammalian models. We thank to the reviewer for his/her relevant comment, that prompted us to find out these differences in the PTEN amino acid sequence from some vertebrate model organisms affecting the BA226 and 425A epitopes.

**Reviewer #2**

Specific points from Reviewer #2:

*1) The term “mAb” (in plural) is used without definition. Please define it.*

*2) The symbol “X” (as in “S229X”) is used without definition. Please define it.*

*3) Page 2, line 8: The phrase “a bias in the of analysis” does not make sense.*

*4) Page 12, line 11, “as well”: Should this be “as well as”?*

*5) Page 12, line 22, “PTEN C-terminal - - -, where also recognized ---”: This sentence does not make sense to me. If the “where” is a typo of “were”, then the sentence makes sense but is logically incorrect, since what were recognized were the proteins and the not mutations. I would therefore put it like, “PTEN with C-terminal truncations ---”.*

*6) Although I am not a native English speaker, usage of the following words/phrases sounds strange to me.*

*i) The “more” on page 3 (line 19) and page 14 (lines 3 and 24) would raise a question “more than what?”. I would use “most” (or try to eliminate “more” when appropriate).*

*ii) “similar than” (page 13, line 9; page 14, line 28): “similar to” sounds better to me.*

1-6) We thank to the reviewer for all the dedicated corrections, which have been addressed in the revised manuscript, including a slight change in the title to avoid using “mAb”.

**Reviewer #3**

Specific points from Reviewer #3:

*Major concerns:*

*1. Figure 1D: Was plasmid transfection efficiency 100%? How long after plasmid transfection were cells obtained for IF experiment? Could authors add negative control?*

The experiments from Figure 1D (and from the new Figure 5C) are transient ectopic expression experiments on COS-7 cells, whose transfection efficiency is about 20%. Non-transfected cells show their nuclei but no PTEN staining, which is in fact a negative control. The cells were processed for immunofluorescence 48 h after transfection, and this has now been indicated more clearly in the Materials and Methods section

*2. Figure 1E: Could authors add commercial PTEN mAb as a positive control? Authors should include sample information (e.g., sample type).*

New Figure 1E now includes comparative analysis of the commercial anti-PTEN 6H2.1 mAb, and sample information has been included in the Figure legend

*3. Figure 2: The principle of truncated forms of PTEN should be described in the manuscript.*

The truncated forms of PTEN used in Figure 2 are now described in the manuscript (page 6), as well as in the Figure legend.

*4. A Δ226-239 PTEN protein was performed in figure 2D, but Δ270-289 is lacking in figure 2E. Could authors explain this?*

We agree with the reviewer that Δ270-289 would be a good control for 425 mAb, but since the 425A mAb has been previously described, we gave priority to the controls regarding the novel BA226 mAb. Nevertheless, the combination of the results using truncation mutations (Figure 2) and amino acid substitutions (Tables 2 and 4) provides a reliable mapping of the 425A epitope.

*5. Please add a statistical method for calculating reactivity of BA226 and 425A.*

The reactivity of BA226 and 425A with the different PTEN variants was determined by visualization from at least two independent experiments, with similar results. For determination, PTEN variants were always run in parallel with PTEN wild type, and reactivity was determined as similar to PTEN wild type (+), diminished reactivity (+/-), or lack of reactivity (-), taking into consideration the expression levels of each variant as indicated by a reference mAb. This has now been indicated under the Materials and Methods section (page 5), as well as in the legends for Tables 1-4

*6. Figure 5B: Please add anti-GAPDH.*

As requested, anti-GAPDH blots have been added to Figure 5B

*Minor concerns:*

*1. Figure 1C: Could authors add concentration of protein?*

As requested, protein concentration has been indicated in the legend to Figure 1C

**Reviewer #4**

Specific points from Reviewer #4:

*This is a well written paper (with only one typo, line 7 of abstract which requires deletion of redundant text “of”) describing the justification and the process follow in creating two antibodies that recognising two sites on the PTEN molecule one of which is not detectable by most agents currently available.*

We thank to the reviewer for the positive comments, and the typo from the Abstract has been amended, as indicated.

*With 6 figures and 4 tables, the authors demonstrate the process undertaken to prove the specificity, though do not test an array of other non-related controls to rule out the problem of cross reactivity.*

Following the reviewer comment, we have added some additional information in the Discussion section (page 15) regarding potential cross-reactivity, as well as cross-species reactivity of the mAb described in our study.

*Also they do not identify what clinical or research question will immediately benefit from understanding of the complexity they have identified*

We have extended the description and make more emphasis on the potential benefits and use in the clinics and in research of the novel anti-PTEN mAb described in our study [Abstract, Results section (pages 6-7), Discussion section (pages 14, 16)].

**Editor comments**

*Please state what role the funders took in the study. If the funders had no role, please state: "The funders had no role in study design, data collection and analysis, decision to publish, or preparation of the manuscript." If this statement is not correct you must amend it as needed.*

*Please include this amended Role of Funder statement in your cover letter; we will change the online submission form on your behalf.*

The indicated statement has been added to the new Funding section.

*PLOS ONE now requires that authors provide the original uncropped and unadjusted images underlying all blot or gel results reported in a submission’s figures or Supporting Information files.*

Uncropped files are provided as a pdf file in the Supported Information.

*In your Data Availability statement, you have not specified where the minimal data set underlying the results described in your manuscript can be found.*

A Data Availability statement has been added.

We thank again to the Editor and to the reviewers for their time and dedicated review and comments, and for their support and suggestions to improve our manuscript. We hope that the revised version of the manuscript will be suitable for publication in PLOS ONE.

Sincerely,

Rafael Pulido

Biocruces Bizkaia Health Research Institute
